# The Concept of Stroma AReactive Invasion Front Areas (SARIFA) as a new prognostic biomarker for lipid-driven cancers holds true in pancreatic ductal adenocarcinoma

**DOI:** 10.1186/s12885-024-12519-9

**Published:** 2024-06-26

**Authors:** Przemyslaw Grochowski, Bianca Grosser, Florian Sommer, Andreas Probst, Johanna Waidhauser, Gerhard Schenkirsch, Nic G. Reitsam, Bruno Märkl

**Affiliations:** 1 Pathology, Faculty of Medicine, University of Augsburg, Augsburg, Germany; 2 General, Visceral and Transplantation Surgery, Faculty of Medicine, University of Augsburg, Augsburg, Germany; 3 Gastroenterology, Faculty of Medicine, University of Augsburg, Augsburg, Germany; 4 Hematology and Oncology, Faculty of Medicine, University of Augsburg, Augsburg, Germany; 5 Tumour Data Management, University Hospital Augsburg, Augsburg, Germany

**Keywords:** PDAC, SARIFA, Lipid metabolism, Pancreas

## Abstract

**Background:**

Pancreatic ductal adenocarcinoma (PDAC) is a ‘difficult-to-treat’ entity. To forecast its prognosis, we introduced a new biomarker, SARIFA (stroma areactive invasion front areas), which are areas at the tumour invasion front lacking desmoplastic stroma reaction upon malignant invasion in the surrounding tissue, leading to direct contact between tumour cells and adipocytes. SARIFA showed its significance in gastric and colorectal carcinoma, revealing lipid metabolism alternations that promote tumour progression.

**Methods:**

We reviewed the SARIFA status of 166 PDAC cases on all available H&E-stained tumour slides from archival Whipple-resection specimens. SARIFA positivity was defined as SARIFA detection in at least 66% of the available slides. To investigate alterations in tumour metabolism and microenvironment, we performed immunohistochemical staining for FABP4, CD36 and CD68. To verify and quantify a supposed delipidation of adipocytes, adipose tissue was digitally morphometrised.

**Results:**

In total, 53 cases (32%) were classified as SARIFA positive and 113 (68%) as SARIFA negative. Patients with SARIFA-positive PDAC showed a significantly worse overall survival compared with SARIFA-negative cases (median overall survival: 11.0 months vs. 22.0 months, HR: 1.570 (1.082–2.278), 95% CI, *p* = 0.018), which was independent from other prognostic markers (*p* = 0.014). At the invasion front of SARIFA-positive PDAC, we observed significantly higher expression of FABP4 (*p* < 0.0001) and higher concentrations of CD68^+^ macrophages (*p* = 0.031) related to a higher risk of tumour progression. CD36 staining showed no significant expression differences. The adipocyte areas at the invasion front were significantly smaller, with mean values of 4021 ± 1058 µm^2^ and 1812 ± 1008 µm^2^ for the SARIFA-negative and -positive cases, respectively (*p* < 0.001).

**Conclusions:**

SARIFA is a promising prognostic biomarker for PDAC. Its assessment is characterised by simplicity and low effort. The mechanisms behind SARIFA suggest a tumour-promoting increased lipid metabolism and altered immune background, both showing new therapeutic avenues.

**Supplementary Information:**

The online version contains supplementary material available at 10.1186/s12885-024-12519-9.

## Background

Worldwide, pancreatic cancer is the fourteenth most common malignancy but ranks seventh in cancer-related deaths [1] and is even prognosed to become the second most common cancer-related cause of mortality by 2030 [2]. The therapy still mainly relies on surgery (Whipple procedure) and adjuvant chemotherapy. However, in 85–90% of cases, tumours are primarily unresectable because of the infiltration of neighbouring structures or the presence of distant metastases [3]. Therapeutic improvements over the past two decades have been limited, and the disease is rightly described as a ‘difficult-to-treat’ entity with a five-year survival rate of only 11% [4]. Compared with other entities such as breast or lung cancer, there are only a few widely accepted prognostic factors routinely implemented in pathological diagnostic workups, including the factors of tumour-node-metastasis (TNM) classification, microsatellite instability status [5] and BRCA mutational analyses [6]; hence, there is a lack of further established and routinely applicable markers.

In our recent studies on gastric and colon adenocarcinomas [7, 8], we established a new histomorphological biomarker called SARIFAs (stroma areactive invasion front areas), which proved to be of independent prognostic relevance in these entities. Also in prostate cancer, a prognostic value could be demonstrated [9]. By definition, a SARIFA is characterised as an area at the tumour invasion front where there is an absence of desmoplastic stroma reaction on malignant invasion in the surrounding inobtrusive tissue, hence leading to direct contact between tumour cells and adipocytes. Detectable on haematoxylin and eosin (H&E)–stained slides, without the necessity for additional immunohistochemistry, simple to learn, and assessable in a short period with low interobserver variability, SARIFAs can be easily implemented in routine diagnostic workflow [7, 8].

Moreover, SARIFA positivity potentially reflects metabolic reprogramming in which tumour cells gain advantage from enhanced lipid supply, as a part of lipidomic remodeling which accompanies malignant transformation [10]. The access to exogenous lipid acids can be obtained through elevated expression of transport proteins including CD36, also known as fatty acid translocase, which with high affinity binds lipoproteins [11] and has been shown to be a negative prognostic marker e. g. in ovarian cancer, enabling the tumour cells a direct uptake of long chain fatty acids from neighboring adipocytes [12]. An alternative pathway of transportation of saturated and unsaturated lipids and fatty acids between tumour cells and adipocytes is conducted through fatty acid binding protein 4 (FABP4) [13, 14]. Both CD36 and FABP4 were shown to be upregulated in SARIFA-positive colorectal and gastric carcinomas [8, 15], an observation suggesting an altered lipid metabolism, which is a promising target for the development of new therapy concepts [16].

Our previous observations led to the question of whether SARIFAs also occur in pancreatic ductal adenocarcinoma (PDAC), an entity known for its pronounced stromal desmoplastic component, and if this concept could be adapted for a neoplasm with a considerably different biology compared with the originally addressed ones.

Therefore, we hypothesised that this phenomenon (i) also occurs in PDAC, (ii) is significantly prognostic and (iii) shows signs of an enhanced lipid metabolism. To confirm these hypotheses, we conducted the first analysis of a local PDAC patient collective and additionally explored the biochemical and immune background via immunohistochemistry.

## Methods

### Patient cohort and ethical approval

The study collection consisted of 166 patients who underwent the Whipple procedure at the University Medical Centre Augsburg between 2005 and 2015. The inclusion criteria were a postoperative survival of > 30 days and histologically confirmed diagnosis of PDAC in the resection specimen. Histopathological diagnoses other than PDAC, incomplete data on staging or death within the first 30 postoperative days led to exclusion.

Staging was performed according to the 8th Union for International Cancer Control staging system [17], grading according to WHO system [18], R-status was assessed according to the criteria proposed by Esposito et al. [19]. Both intrapancreatic and retroperitoneal resection margins were considered. The sample size was not statistically determined prior to investigation.

Histologic subtyping was not investigated. Because of the limited number of cases, a division between test and validation collections, as recommended by REMARK [20] and STROBE [21] guidelines, could not be conducted. The study was performed in compliance with the Declaration of Helsinki. The protocol was evaluated and approved by the ethical committee of the Ludwig Maximilian University of Munich (reference: 22–0437), with no declaration of consent from the patients required.

The clinical data were derived from Tumour Data Management, University Hospital of Augsburg, and completed with the information acquired from the patient files. The gathered data included: age at diagnosis, sex, adjuvant therapy, local recurrence and/or distant metastasis, last recorded medical contact or for deceased patients date of death, and in some cases additionally body mass index (BMI). The endpoint of the study was overall survival (OS), which was measured from the moment of diagnosis to death of any cause or last registered follow-up (censored entries). The median follow-up was calculated using the reverse Kaplan–Meier method [22]. The estimated median follow-up for the whole study collection was 78.2 months (66.5–113.5) and did not differ significantly between SARIFA-positive and -negative cases (*p* = 0.405).

The power of our survival analysis was calculated with the R-package *‘powerSurvEpi’.* The power of our study was moderate with 0.685 (sample size *n* = 166, hazard ratio for SARIFA-positivity on OS from univariate analysis 1.56, significance level 0.05, event rate 0.75).

### Histopathological SARIFA assessment

**Fig. 1:**
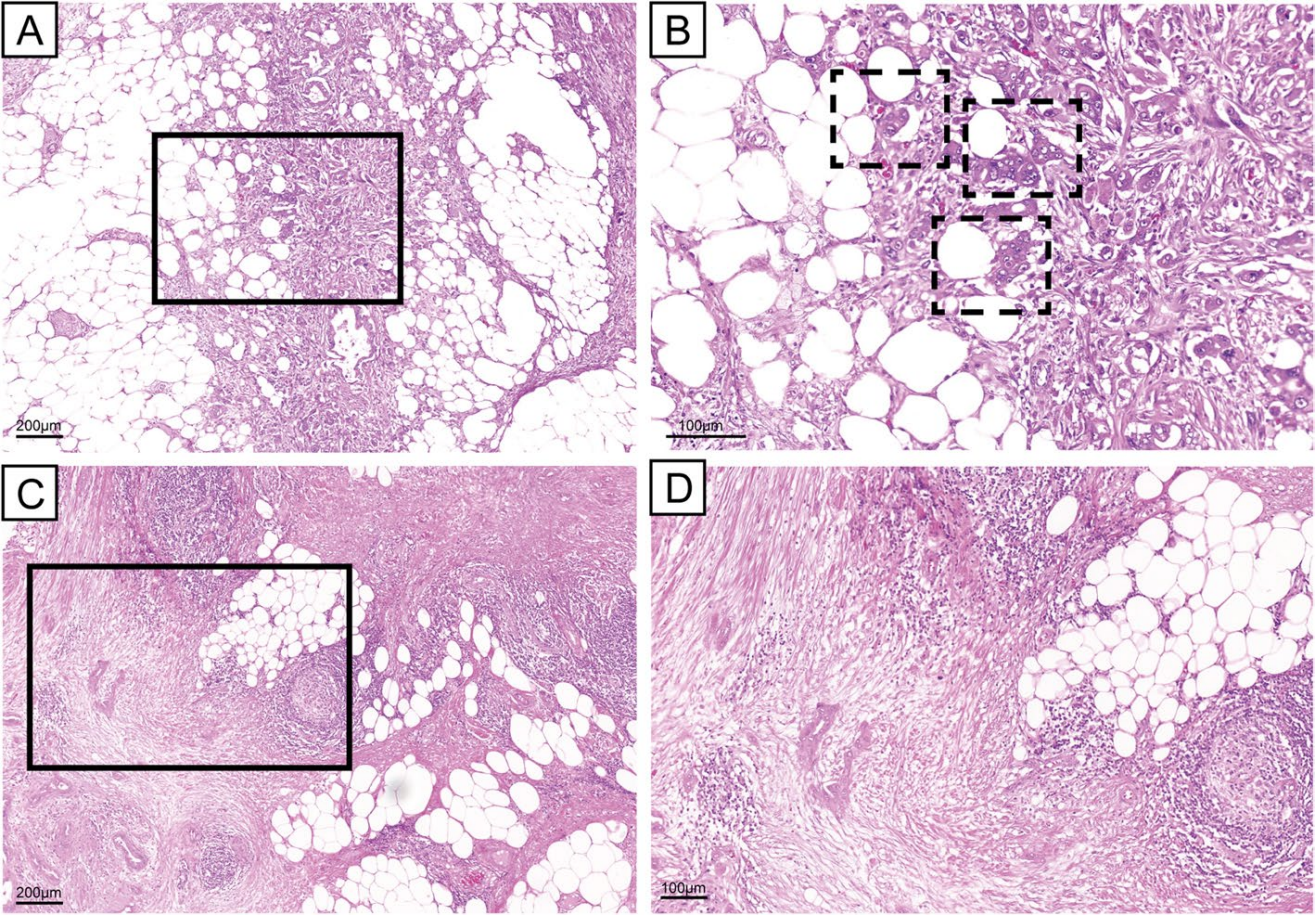
SARIFA-positive and -negative cases; H&E staining. **A** Exemplary SARIFA-positive PDAC with tumorous cells directly adjacent to adipocytes at the invasion front; scale bar 200 µm. **B** Detailed picture of SARIFAs (marked with a dashed line); scale bar 100 µm. **C** SARIFA-negative PDAC with desmoplastic tumorous stroma separating malignant cells from surrounding fatty tissue; scale bar 200 µm. **D** Detailed picture of SARIFA-negative PDAC; H&E; scale bar 100 µm. SARIFA – stroma areactive invasion front area; PDAC – pancreatic ductal adenocarcinoma

All given H&E-stained tumour slides (total 931, median 5 per case), each covering an area of approximately 220 mm^2^, were examined by two independent investigators (PG and BM) who were blinded to the clinicopathological data. A SARIFA was defined as the direct contact between at least five tumorous cells or a malignant gland and inconspicuous adipocytes at the invasion front or within the pancreas, as described recently by our group [7, 8]. Due to its histologic structure, there is also intraparenchymal adipose tissue in the pancreas. As we believe that direct tumour-adipocyte interactions represent an underlying tumour biology, we also considered this tumour-adipocyte contact as SARIFA-positivity—even though it is not at the invasion front. For consistency with our previous publications, we still refer to this as SARIFA. Representative images of both SARIFA-positive and SARIFA-negative cases are presented in Fig. 1. Because the morphological feature of a SARIFA itself occurs at a high frequency in PDAC and not only at the invasion front, we decided to renounce the restriction of the invasion front and counted also intra-parenchymal interactions with adipocytes. Moreover, we established a quantitative cut-off for classifying a case as SARIFA-positive based on the number of SARIFA-positive slides within each PDAC case, similarly to our study on SARIFA in prostate cancer [9]. For cut-off calculation, we used the R-package ‘bhm’ [23, 24]. The estimated optimal cut-off threshold based on the percentage of slides showing SARIFA was 0.6680 (95% confidence interval: 0.5842—0.711). By using this cut-off, we reached a SARIFA-positive frequency of 31.9%, which is similar to the SARIFA frequency in colorectal and gastric cancer [8, 15, 25]. For more details on our biomarker cut-off, refer to Figure S1. Following the independent assessment by two investigators, the cases with discrepant SARIFA scores were re-evaluated jointly by the same investigators, and a consensus diagnosis was made using a double-headed microscope.

### Immunohistochemical studies

Additional immunohistochemical staining was performed to analyse and compare the expression of fatty acid metabolism–related proteins and the role of macrophages in SARIFAs, here corresponding to the results of preceding analyses on gastric carcinoma [8]. FABP4, CD36 and CD68 immunohistochemistry was performed on 30 SARIFA-positive and 30 SARIFA-negative representative cases, using 2- to 4-µm-thick, whole-slide, formalin-fixed paraffin-embedded sections. The staining was performed on a Leica Bond RX automated staining system (Leica, Wetzlar, Germany) according to the automated immunohistochemical protocol optimised for use on this platform (antibodies and dilution in Supplementary Information Table S1). The assessment of FABP4 and CD36 at both the invasion front and tumour centre was conducted using the immunoreactive score, which is a seven-tier semiquantitative scoring system, as proposed by Remmele and Stegner [26]. Therefore, staining intensity and the percentage of positive tumour cells were evaluated to calculate the score accordingly. The number of CD68-positive macrophages was counted on a representative high-power field at the tumour centre and invasion front. Representative areas at the invasion front and tumour centre were selected by visual impression.

### Adipocyte morphometry

**Fig. 2:**
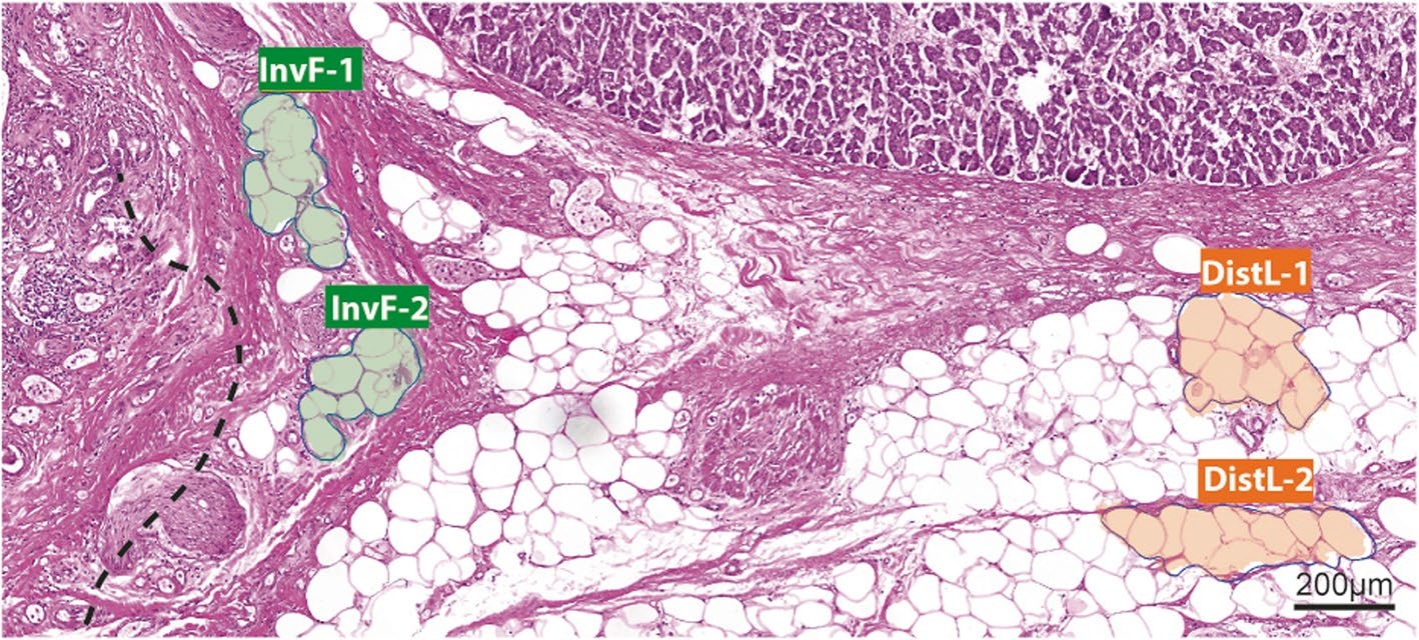
Principle of morphometric measurement of adipocytes. Exemplary PDAC slide with marked adipocytes at invasion front (InvF-1 and -2) and in distant locations (DistL-1 and -2), each with approx. 10 adipocytes; H&E; scale bar 200 µm. PDAC – pancreatic ductal adenocarcinoma

To verify and quantify a supposed delipidation of adipocytes, areas were digitally morphometrised. For that, H&E slides of 10 randomly selected SARIFA-positive and 10 SARIFA-negative cases from the above-described immunohistochemistry cohort were scanned using a 3D Histech Panoramic Scan II (3D Histech, Budapest, Hungary), and the morphometric measurements were performed using the CaseViewer 2.4 software (3DHistech, Budapest, Hungary). Two adipocytic areas each of the invasion front and of locations distanced at least 1 mm from the tumour were analysed by one investigator (BM) by measuring the area of 4 to 13 adipocytes (mean: 10 ± 2) (Fig. 2).

### Statistical analysis

SPSS version 29.0 (SPSS, IBM, Chicago, IL, USA) and RStudio 2022.07.0 (R Foundation for Statistical Computing, Vienna, Austria) were used for statistical analysis. Correlations between clinicopathological data and SARIFA status were tested using Chi-squared tests or Fisher’s exact tests. The Kaplan–Meier method was used to depict the survival rates and the log-rank test to prove the significance of survival between the tested groups. The assessment of interobserver agreement was measured using kappa statistics. Relative risks were estimated by hazard ratios (HRs) calculated via Cox proportional hazard models.

Neither large language models nor artificial intelligence solutions were used in conducting the study.

## Results

### Clinicopathological characteristics of the cohort

In the examined population of 166 PDACs, 21 patients were diagnosed with a primary tumour (pT) in pT1 stage 108 with a pT2 stage and 37 with a pT3 stage. 123 patients presented nodal and 95 distant metastases (seven during surgery, on suspicion of intraoperatively detected abdominal lesions). A total of 112 (78%) patients received adjuvant chemotherapy (CTx) with different treatment regimens: Here, 74 were treated primarily with gemcitabine in monotherapy in a standard scheme of six courses and two with FOLFIRINOX schema. The remaining 36 patients received chemotherapy in other regimens (e.g., gemcitabine combined with erlotinib or radiotherapy) or did not complete the full treatment.

The median age at diagnosis was 68 years (range 44 to 85 years).

### SARIFA in PDAC and correlation with clinicopathological characteristics

Overall, 53 cases (32%) were classified as SARIFA positive and 113 (68%) as SARIFA negative. SARIFA positivity was significantly associated with a higher rate of vascular invasion (*p* = 0.029) and lower frequency of adjuvant therapy (*p* = 0.009).

Other characteristics, including extension of pT, lymph node metastasis (pN) or distant metastasis, and R-status were not associated with SARIFA status (each *p* > 0.05). Detailed clinicopathological data are summarised in Table 1.

**Table 1:** Clinicopathological characteristics

Variable	*n* = 166		SARIFA-positive (*n* = 53)		SARIFA-negative (*n* = 113)		*p*-value
Median age (range), years	68.0 (44–85)		67.5 (45–83)		65.8 (44–85)		0.285
Sex							0.388
Female	77	46%	22	41%	55	49%	
Male	89	54%	31	59%	58	51%	
pT status							0.674
pT1	21	13%	8	15%	13	11%	
pT2	108	65%	35	66%	73	65%	
pT3	37	22%	10	19%	27	24%	
pN status							0.782
Negative	43	26%	13	24%	30	26%	
Positive	123	74%	40	76%	83	74%	
cM							0.911
No	71	43%	23	43%	48	42%	
Yes	95	57%	30	57%	65	58%	
Histological Grading							0.155
G1	12	7%	2	4%	10	9%	
G2	106	64%	31	58%	75	66%	
G3	48	29%	20	38%	28	25%	
Vascular invasion							**0.029**
Negative	135	81%	38	72%	97	86%	
Positive	31	19%	15	28%	16	14%	
Lymphovascular invasion							0.455
Negative	110	66%	33	62%	77	68%	
Positive	56	34%	20	38%	36	32%	
Perineural invasion							0.207
Negative	24	15%	5	9%	19	17%	
Positive	142	85%	48	91%	94	83%	
R status							0.734
R0	72	43%	24	45%	48	43%	
R1	94	57%	29	55%	65	57%	
Local recurrence (*n* = 85)							0.526
Negative	5	6%	1	4%	4	7%	
Positive	80	94%	27	96%	53	93%	
aCTx (*n* = 144)							**0.009**
No	32	22%	16	36%	16	16%	
Yes	112	78%	29	64%	83	84%	

*CI* Confidence interval, *pT* Pathological assessment of extension of primary tumour (according to the 8th UICC staging system), *pN* Pathological assessment of lymph node metastasis (according to the 8 ^th^ UICC staging system), *cM* Clinical assessment of distant metastases, *R* Residual tumour, *aCTx* Adjuvant (in all schemes)

*P* values are shown for differences between SARIFA-positive and SARIFA-negative tumours; bold marked values are statistically significant with *p* < 0.05. Regarding local recurrence (*n* = 85) and adjuvant chemotherapy (*n* = 144), data were only available for a subgroup of patients. For all other parameters, the whole cohort (*n* = 166) was considered.

Because obesity has previously shown significant correlations with the alternation of PDAC cell metabolism towards higher fatty acid uptake and a higher rate of tumour progression, we compared the body mass index between positive and negative patients (30 cases each) and found no significant correlation with the SARIFA status (*p* = 0.32; corresponding boxplot in Supplementary Information Figure S2).

### Interobserver variability

Considering distinctive stromal desmoplasia in PDAC, the assessment of SARIFA status appeared to be a demanding task. Nevertheless, the interobserver variability between the first and last author corresponded with a kappa value of 0.56, showing moderate interobserver agreement.

### Survival analysis

**Fig. 3:**
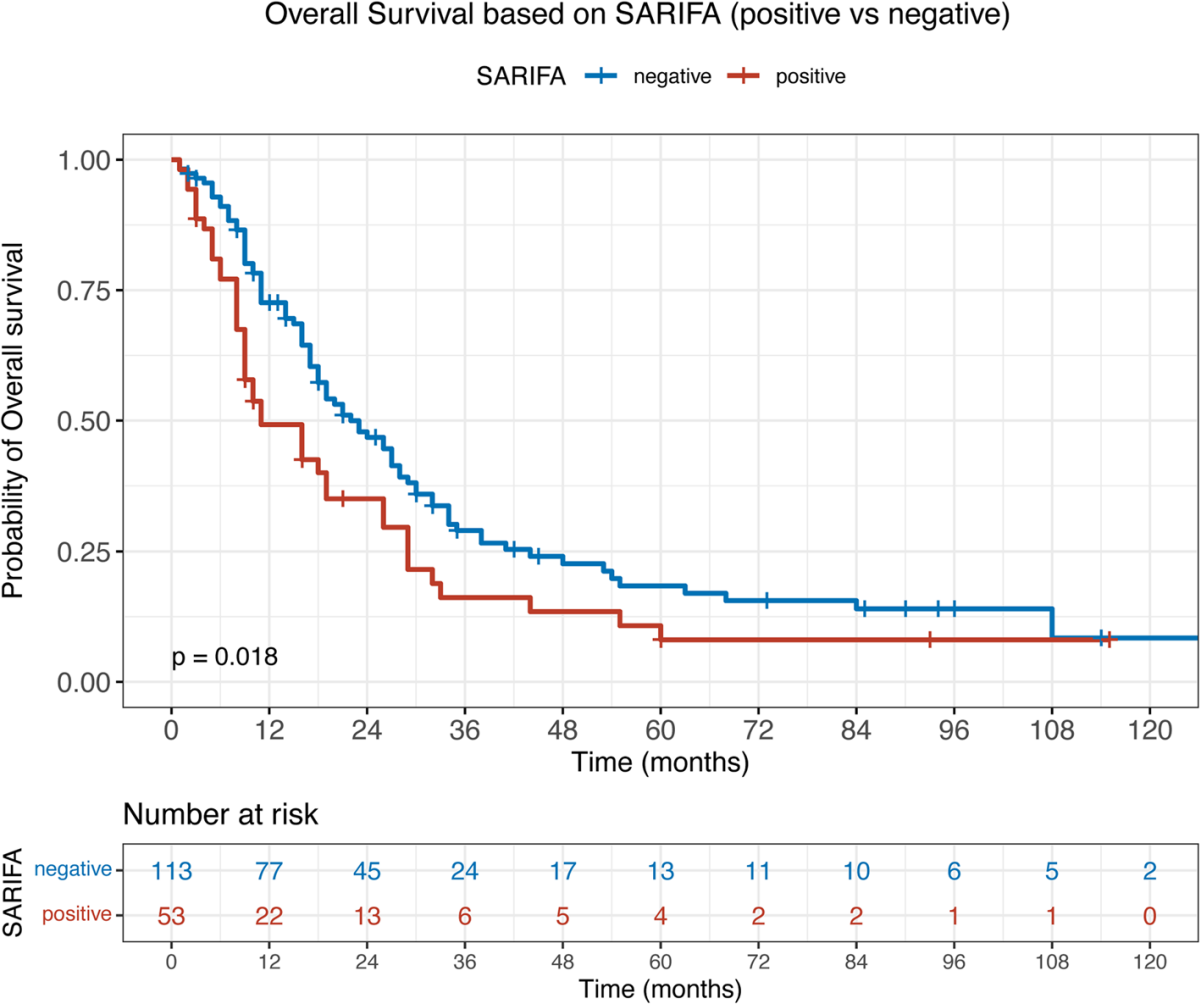
PDAC patient survival dependency on SARIFA status. Kaplan–Meier curve of patients with SARIFA-positive and SARIFA-negative PDAC. *P* value of the log-rank test. SARIFA – stroma areactive invasion front areas; PDAC – pancreatic ductal adenocarcinoma

To analyse the prognostic relevance of SARIFA status in PDAC, we performed a Kaplan–Meier analysis and observed a distinct separation of survival curves (Fig. 3, log-rank, *p* = 0.018). Our analyses showed that patients with SARIFA-positive PDAC had a significantly worse OS compared with SARIFA-negative cases (median OS: 11.0 months vs. 22.0 months, HR: 1.570, 95% CI 1.082–2.278, *p* = 0.018).

To assess the prognostic relevance of SARIFA status compared with other risk factors, we performed uni- and multivariate Cox regression analyses. In the univariate analysis, patients’ age, tumour grading, adjuvant chemotherapy and SARIFA status were significantly related to worse OS (Table 2). In the multivariate analysis, the following common risk factors were included: tumour grading, pT category, lymph node metastasis and invasion in blood or lymphatic vessels. Besides grading, SARIFA status remained significantly associated with shorter OS (*p* = 0.014), indicating that SARIFA status was an independent risk factor (Table 2).

**Table 2:** Uni- and multivariate Cox regression analysis regarding overall survival

	Univariate Cox Regression		Multivariate Cox Regression adjusted for^a^	
	Hazard Ratio (95% CI)	*p-*value	Hazard Ratio (95% CI)	*p-*value
**Overall survival**				
SARIFA	1.570 (1.082–2.278)	**0.018**	1.637 (1.103–2.429)	**0.014**
Age	1.032 (1.012–1.053)	**0.002**		
Grading	2.159 (1.553–3.000)	** < 0.001**	2.042 (1.465–2.847)	** < 0.001**
Sex	0.976 (0.687–1.386)	0.891		
L	1.089 (0.747–1.588)	0.659	1.003 (0.676–1.489)	0.987
V	1.447 (0.926–2.262)	0.105	1.256 (0.787–2.004)	0.340
n	1.386 (0.829–2.318)	0.213		
pT	1.241 (0.922–1.672)	0.154	1.255 (0.902–1.747)	0.178
pN	1.190 (0.930–1.522)	0.166	1.185 (0.926–1.517)	0.178
Adjuvant therapy	0.313 (0.202–0.485)	** < 0.001**		
cM	0.881 (0.619–1.256)	0.484		
R	1.312 (0.919–1.873)	0.135		

*CI* Confidence interval, *L* Lymphovascular invasion, *V* Vascular invasion, *Pn* Perineural invasion, *pT* Pathological assessment of extension of primary tumour (according to the 8th UICC staging system), *pN* Pathological assessment of lymph node metastasis (according to the 8th UICC staging system), *cM* Distant metastasis, *R* Residual tumour

^a^The multivariate model was adjusted for SARIFA, grading, pT, pN, L, and V

To assess the effect of SARIFA status on the impact of adjuvant therapy and, hence, whether SARIFA status may be predictive, we performed further subgroup analyses. A significantly lower percentage of SARIFA-positive PDAC patients received adjuvant therapy compared to SARIFA-negative PDAC patients (64.4% vs. 83.8%, *p* = 0.009). In SARIFA-positive PDACs, adjuvant therapy was significantly associated with better OS (HR: 0.344, 95% CI 0.171–0.692, *p* = 0.002) but with a limited number of included patients (n total: 45, adjuvant therapy *n* = 29, no adjuvant therapy *n* = 16). This was also true within SARIFA-negative PDACs (n total: 99, adjuvant therapy *n* = 83, no adjuvant therapy *n* = 16) because adjuvant treatment was again associated with better OS (HR: 0.315, 95% CI 0.176–0.564, *p* < 0.001). These findings show that patients with PDAC benefit from adjuvant chemotherapy regardless of SARIFA-status [27]. Corresponding Kaplan–Meier curves are provided in the Supplementary Information (Figure S3).

### Immunohistochemical expression of FABP4, CD36 and CD68 at SARIFAs

**Fig. 4:**
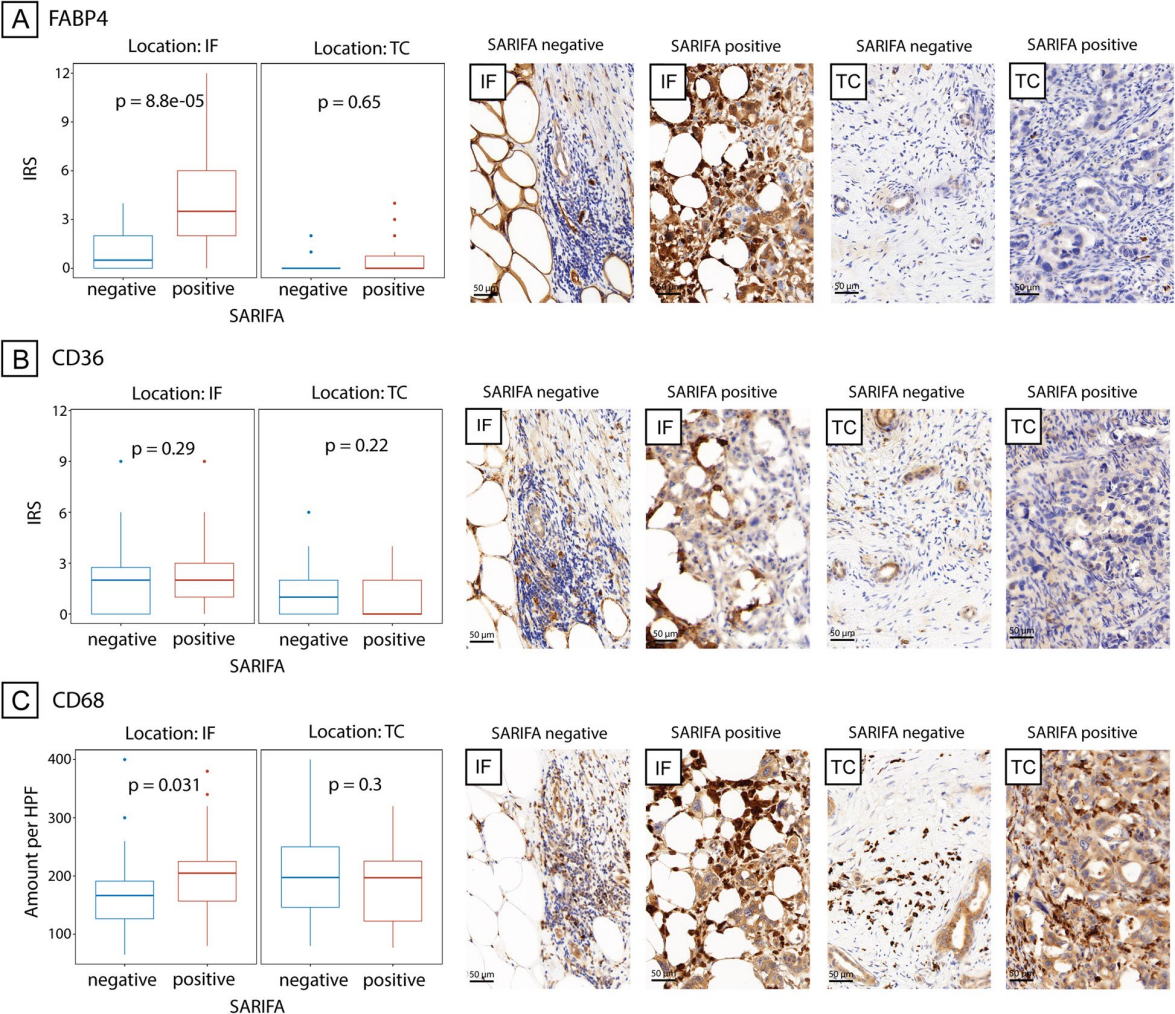
Expression of FABP4, CD36 and the presence of CD68-positive macrophages in SARIFA-positive and -negative cases at the tumour centre and invasion front. Boxplot showing differences in **A** FABP4 expression, **B** CD36 expression, and **C** CD68 + macrophage count at invasion front (IF) and tumour centre (TC) with exemplary images. SARIFA – stroma areactive invasion front area; IRS – immunoreactive score; HPF – high-power field. Scale bar 50 µm

As mentioned above, we completed additional immunohistochemical studies focusing on lipid metabolism and tumour-associated macrophages at SARIFAs. Therefore, we investigated FABP4 and CD36 expression and the number of CD68^+^ macrophages. Tumour cells in SARIFA-positive cases showed higher expression of FABP4 at the invasion front than in SARIFA-negative cases (*p* < 0.0001). CD36 expression showed no statistically significant SARIFA-dependent changes (each *p* > 0.05). Moreover, CD68^+^ macrophages showed a higher density at the invasion front of SARIFA-positive than SARIFA-negative PDACs (*p* = 0.031). In SARIFA-negative regions, no differences regarding FABP4 and CD36 expression, as well as CD68^+^ macrophages, could be found (each *p* > 0.05). Immunohistochemical stains and the corresponding results are visualised in Fig. 4.

### Adipocyte morphometry

**Fig. 5:**
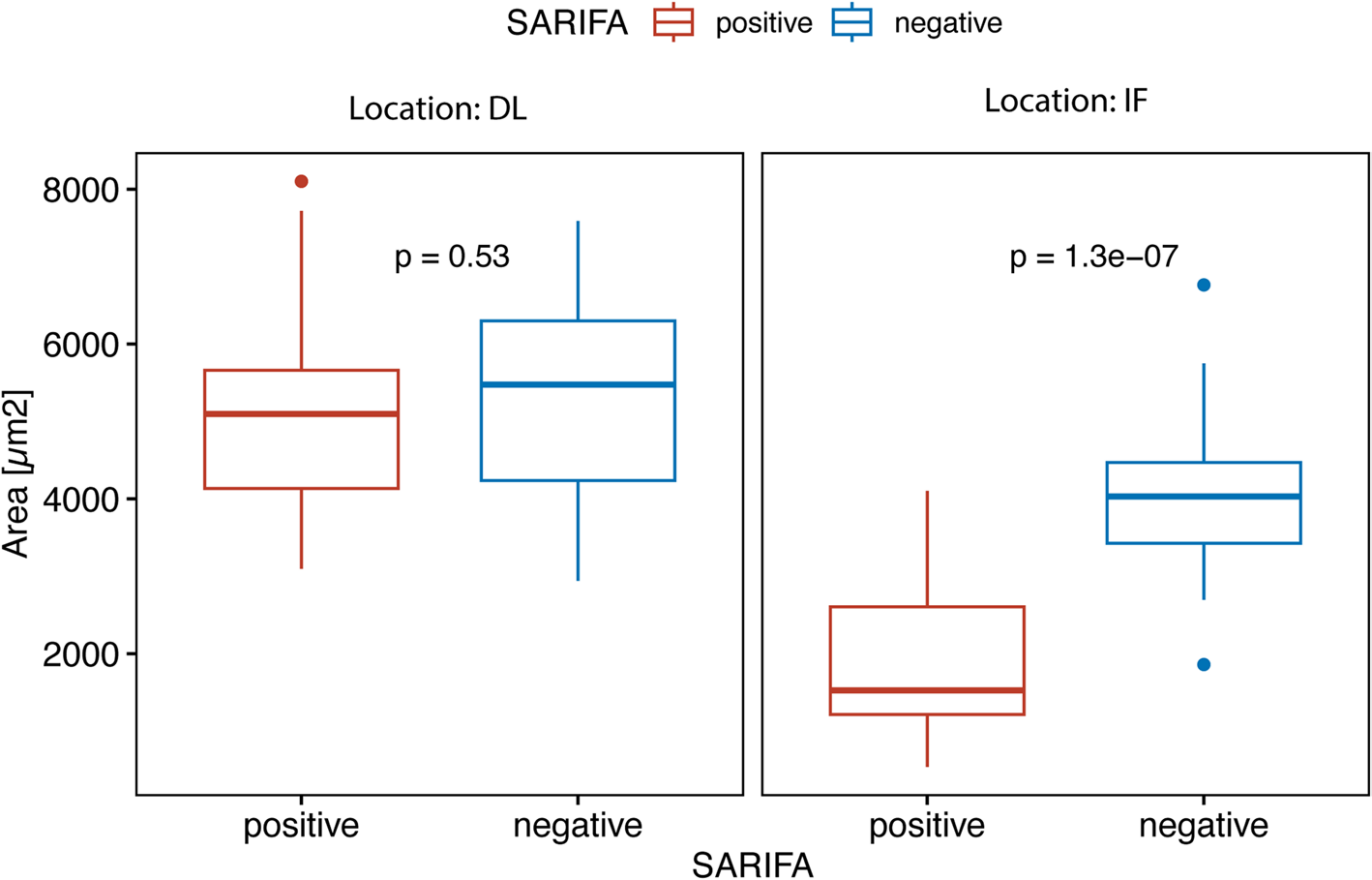
Adipocyte morphometry in SARIFA-positive and -negative cases at invasion front and locations distant from tumour. Boxplot showing differences in size of areas of approx. 10 adipocytes between SARIFA-positive and—negative cases in distant locations (DL) and invasion front (IF); SARIFA – stroma areactive invasion front areas

The adipocyte size in tumour-distanced locations did not differ between SARIFA-negative and -positive cases, with mean values of 5356 ± 1514 µm^2^ and 5140 ± 1559 µm^2^ (*p* = 0.659), respectively. The adipocyte areas at the invasion front were significantly smaller, with mean values of 4021 ± 1058 µm^2^ and 1812 ± 1008 µm^2^ for the SARIFA-negative and -positive cases, respectively. The differences between the SARIFA-positive invasion front adipocyte areas were highly significant (*p* < 0.001) (Fig. 5).

## Discussion

The role of lipid metabolism has gained an increasing recognition in cancer research, as it offers a potential for new therapeutic targets [16]. With SARIFAs, we introduced a new prognostic biomarker whose biological significance lies in reflecting lipid-driven changes of tumour metabolism, so far demonstrated in gastric, colorectal and prostate cancer. In contrast to what might be expected, the occurrence of SARIFAs did not correlate with obesity [8, 9]. In the current study, we tested the hypotheses that SARIFA classification is applicable to and prognostic in PDAC and reveals signs of enhanced lipid metabolism.

Indeed, the SARIFA classification is applicable to PDAC, however, it had to be adapted compared to previous studies on gastric and colorectal cancer. The restriction to evaluate only the invasion front had to be abandoned and a quantitative cut-off had to be established. The evaluation was more challenging compared with the previous applications, resulting in a lower but still acceptable kappa value similar to the range of other experimental histological features [28]. Ongoing research on the classification of PDAC using deep neural networks opens up perspectives for further improvement of the evaluation of SARIFAs and is reassuring regarding the reproducibility of assessment [29].

Compared with other tumours, in PDAC, there is a lack of biomarkers, and the 8th Union for International Cancer Control TNM staging system plays the most important yet debatable role in this context [30, 31], indicating the need for new biomarkers. DNA/RNA-based or subtype analysis and gene expression profiling are cost- and time-consuming assays and currently often have limited availability [5, 32–34].

In line with our findings, several studies deploying deep-learning algorithms on digitised slides of colorectal cancer were able to identify a morphologically similar phenomenon described as a ‘tumour adipose feature’ or ‘adipocytes close to tumour cells’, proven to be prognostically highly relevant [35–37]. These studies support our hypothesis regarding the relevance of direct interactions between tumour cells and adipocytes. This is further strengthened by animal and in vitro PDAC models [38, 39], which have shown that adipocytes interact with and directly promote proliferation of malignant cells by increasing their fat uptake. To verify these findings in context of SARIFA-phenomenon in PDAC in human tissue, we performed immunohistochemistry for CD36 and FABP4, two proteins that play major roles in lipid metabolism. As a result, we were able to demonstrate a significantly increased FABP4 expression, particularly at SARIFAs. The immunohistochemical expression of CD36, a multiligand translocase enabling transmembranous allocation of oxidised low-density lipoproteins, does not differ between SARIFA-positive and SARIFA-negative PDAC. These findings deviate from the analyses by Grosser et al. in gastric cancers, where CD36 expression was more pronounced in SARIFA-positive tumours [8], indicating that the regulation mechanism in these two entities differs. Therefore, the uptake of fatty acid could rely on an alternative transport mechanism like extracellular vesicles [40]. FABP4 is responsible for intracellular transportation and metabolism of fatty acids and was previously reported to be associated with poor prognosis in PDAC [41] and other malignancies [42], which is in line with our findings. Its upregulated expression in the context of direct contact between malignant cells and adipocytes, even in a highly glucose-dependent malignancy such as pancreatic cancer [43], suggests a more distinctive role of fatty acids as an energy source and supply of building blocks for cellular membranes in SARIFA-positive PDAC. The fact that adipocytes shrink when coming into contact particularly with tumour cells, as shown by our morphometric analyses, suggests adipocytes’ delipidation and uptake of lipids by the tumour cells. There is a large body of evidence indicating that lipids play a fundamental role in tumour progression [16, 44]. Metabolic reprogramming has been included in the hallmarks of cancers [45]. It seems likely that SARIFAs could serve as a biomarker that is not only prognostic but also effective for the selection of tumours that are particularly driven by lipids, what on the other hand could pave the way for new treatment approaches specifically targeting lipid metabolism in SARIFA-positive PDAC, for example, by using metformin, CPT1 or FABP4 inhibitors [16, 46–48].

Among the several cell populations influencing both the growth and chemotherapy resistance of PDAC, tumour-associated macrophages drew our attention as an essential component of its microenvironment, playing a significant role in its biology [49, 50]. Moreover, CD68 + macrophages were upregulated at the SARIFAs in our study of gastric cancer [8]. In line with this, we observed higher concentrations of CD68 + macrophages at the invasion front of SARIFA-positive PDAC compared with SARIFA-negative cases, whereas in the tumour centre, there was no difference. Di Caro et al. showed that a higher density of macrophage infiltration at the tumour–stroma interface is associated with progression and distant metastasis of therapy-naïve PDAC [51] as a result of tumour-associated macrophages’ immunosuppressive activity. This mechanism could be co-responsible for both the development of SARIFAs and the non-favourable prognosis of SARIFA-positive PDAC cases, along with other alterations in local immune response [25].

The retrospective nature of the present study and the relatively low case numbers constitute its major limitations. A retrospective investigation of available material is inevitably combined with loss of some information, e. g. on clinical data. In similar retrospective studies, for example on tumor budding in PDAC [52] a cohort of 173 patients was investigated, a similar number comparing to our study. Nonetheless it was not possible to introduce test and validation groups in the study design. Therefore a future prospect study on SARIFA in PDAC would be of unpresented value, providing further aspects of the phenomenon described by us.

For our cohort only overall survival, which is considered the most important survival endpoint in cancer medicine [53], was available. Nevertheless, future studies should include disease-specific and progression-free survival to gain further insights into the prognostic relevance of SARIFA-status in PDAC.

Additional limitations are due to single site and temporal constraint of our study. We investigated a cohort of patients who were treated at our surgical department between 2005 and 2015. A multicenter study design could reassure objectivity of the observations and confirm our findings. Finally incorporating advanced methodologies, such as spatial expression profiling (similarly to earlier studies on SARIFA by Grosser et al.), could provide deeper scope in local tumor environment and contribute to more detailed description of changes in SARIFA, which seem to be of an immune nature [25] and still require further investigation, not only in PDAC but also in other entities.

## Conclusions

The present study has shown first evidence for SARIFA status as a negative prognostic factor in PDAC. Compared with other novel biomarker approaches, which can only partly be evaluated on H&E-stained slides, SARIFA assessment is characterised by its simplicity and low effort, enabling reliable patient stratification. The mechanisms behind SARIFAs suggest the major role of an increased tumour-promoting lipid metabolism and altered immune background. Therefore, we propose SARIFA status as a novel H&E-based biomarker in PDAC that potentially could not only help better stratify patients but also guide new therapeutic avenues by interfering in the lipid metabolism of tumour cells, if subsequent studies build upon our findings.

### Supplementary Information

Supplementary Material 1.

Supplementary Material 2.

Supplementary Material 3.

Supplementary Material 4.

## Data Availability

The datasets generated throughout the analysis can be obtained from the corresponding author upon reasonable request.

## Abbreviations

CI: Confidence interval
CTx: Adjuvant chemotherapy
H&E: Haematoxylin and eosin
HR: Hazard ratio
OS: Overall survival
PDAC: Pancreatic ductal adenocarcinoma
pT: Primary tumour
pN: Lymph node metastasis
SARIFAs: Stroma areactive invasion front areas
TNM classification: Tumour-node-metastasis classification
