# Supplementary material for: The Concept of Stroma AReactive Invasion Front Areas (SARIFA) as a new prognostic biomarker for lipid-driven cancers holds true in pancreatic ductal adenocarcinoma
Source: BMC Cancer. 2024 Jun 26;24:768. doi: 10.1186/s12885-024-12519-9 (PMC11210040; doi:10.1186/s12885-024-12519-9)
Supplement: Supplementary file 1 — Supplementary Material 1. [file 12885_2024_12519_MOESM1_ESM.pdf]

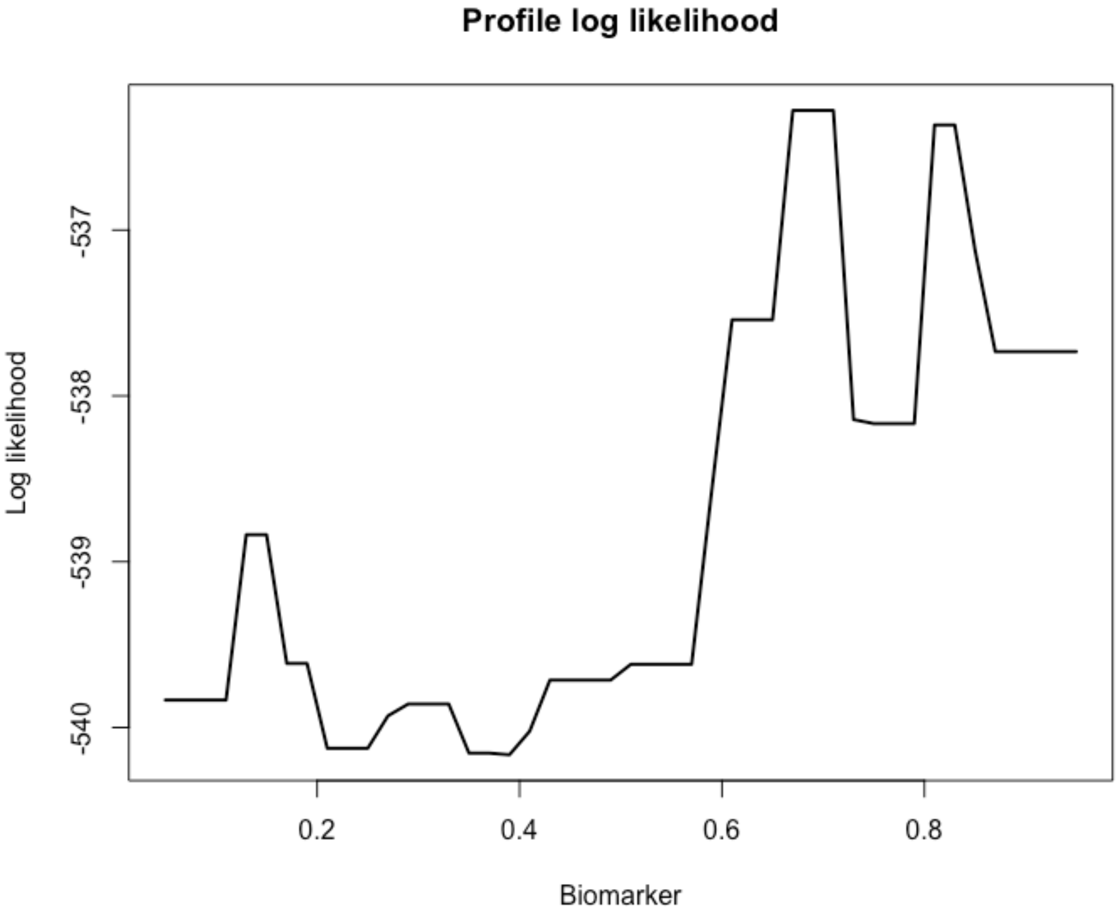

Figure S1. Biomarker threshold regression model for identifying optimal cut-off of SARIFA-positive slides (Biomarker). By using the R package *'bhm'*, we could establish a cut-off of 0.6680 (66.8%) of SARIFA-positive slides for an optimal prognostic discrimination (estimate of biomarker threshold model: 0.6680 with 95% confidence interval of 0.5842-0.711). For practicability, we classified a case as SARIFA-positive when at least 2/3 of all slides showed SARIFA.

Biomarker: in our case, frequency (%) SARIFA-positive slides, SARIFA: Stroma AReactive Invasion Front Areas.
