# Supplementary material for: The Concept of Stroma AReactive Invasion Front Areas (SARIFA) as a new prognostic biomarker for lipid-driven cancers holds true in pancreatic ductal adenocarcinoma
Source: BMC Cancer. 2024 Jun 26;24:768. doi: 10.1186/s12885-024-12519-9 (PMC11210040; doi:10.1186/s12885-024-12519-9)
Supplement: Supplementary file 2 — Supplementary Material 2. [file 12885_2024_12519_MOESM2_ESM.pdf]

Figure S2 BMI differences between patients with SARIFA negative and positive PDAC

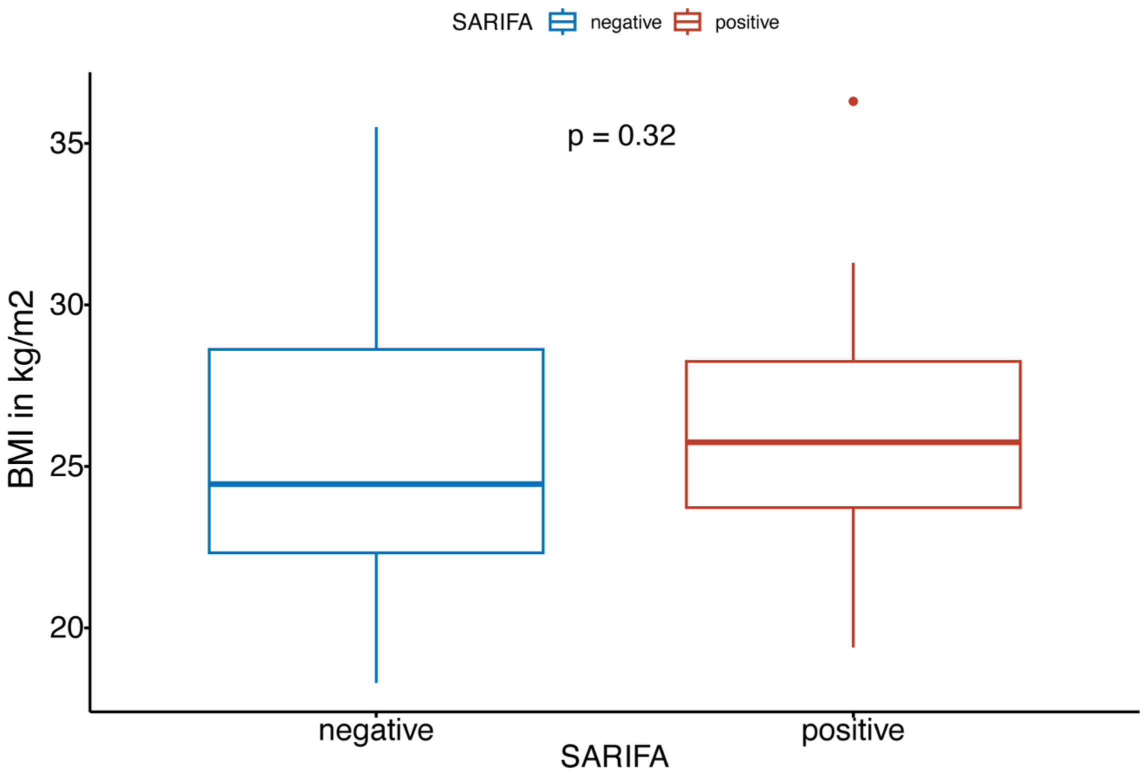

Boxplot showing differences in BMI between patients with SARIFA negative and positive PDAC; BMI – body mass index; SARIFA – stroma areactive invasion front area; PDAC – pancreatic ductal adenocarcinoma.
