# Supplementary material for: The Concept of Stroma AReactive Invasion Front Areas (SARIFA) as a new prognostic biomarker for lipid-driven cancers holds true in pancreatic ductal adenocarcinoma
Source: BMC Cancer. 2024 Jun 26;24:768. doi: 10.1186/s12885-024-12519-9 (PMC11210040; doi:10.1186/s12885-024-12519-9)
Supplement: Supplementary file 3 — Supplementary Material 3. [file 12885_2024_12519_MOESM3_ESM.pdf]

Figure S3 PDAC patient survival dependency on adjuvant therapy in SARIFA positive and negative groups

Overall Survival of SARIFA−positive PDAC stratified by adjuvant therapy

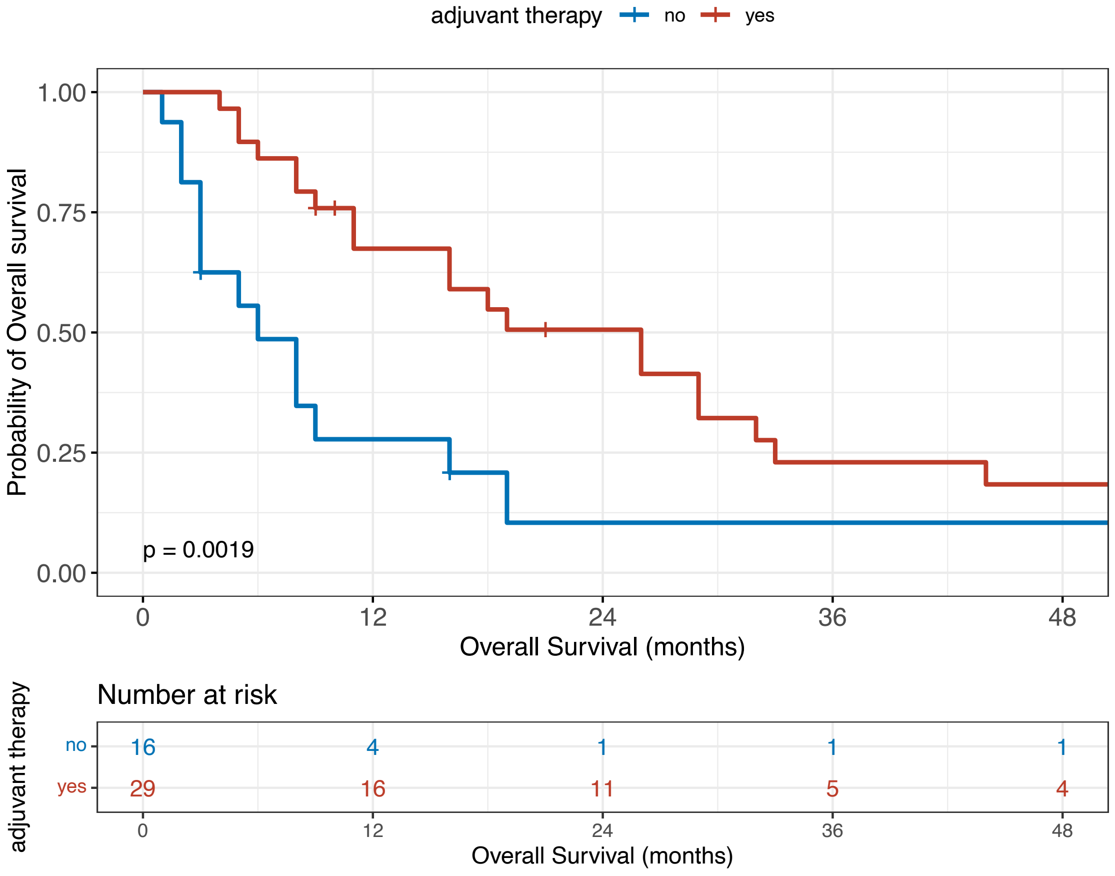

Overall Survival of SARIFA−negative PDAC stratified by adjuvant therapy

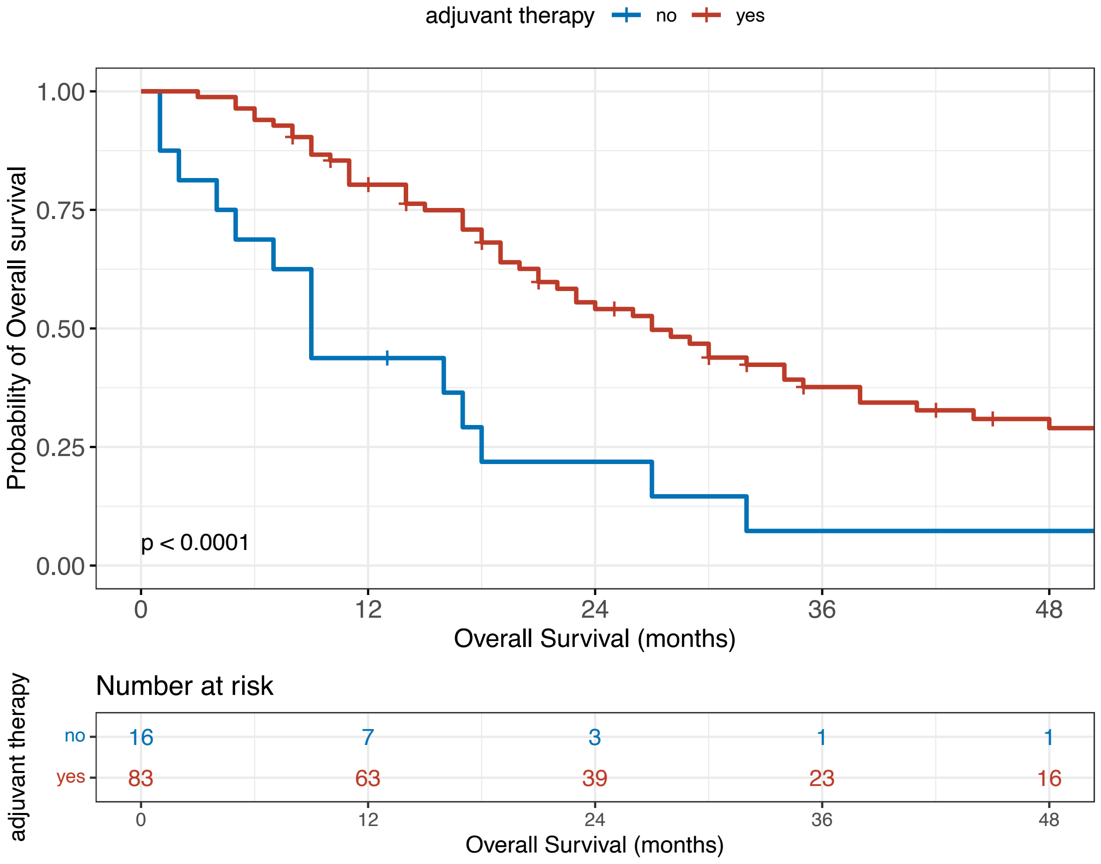

Kaplan–Meier curve of patients with SARIFA-positive and negative PDAC: survival dependency on adjuvant therapy; SARIFA – stroma areactive invasion front area; PDAC – pancreatic ductal adenocarcinoma.
