# Supplementary material for: The Concept of Stroma AReactive Invasion Front Areas (SARIFA) as a new prognostic biomarker for lipid-driven cancers holds true in pancreatic ductal adenocarcinoma
Source: BMC Cancer. 2024 Jun 26;24:768. doi: 10.1186/s12885-024-12519-9 (PMC11210040; doi:10.1186/s12885-024-12519-9)
Supplement: Supplementary file 4 — Supplementary Material 4. [file 12885_2024_12519_MOESM4_ESM.pdf]

Table S1 Antibodies and dilution used for immunohistochemical staining

Antigen	Clone	RRID	Supplier	Dilution	Positive control	Negative control	Cellular location of immunolabel
CD36	HPA002018	AB_1078464	Sigma Aldrich (St Louis, MO, USA)	1:50	Placenta	Internal negative control in the stained tissue and one slide per run omitting the primary antibody	cytoplasmatic
CD68	KP-1	AB_1158192	Cell Marque	1:200	Tonsil		cytoplasmatic
FABP4	ab13979	AB_1951817	Abcam	1:500	Liposarcoma		cytoplasmatic
